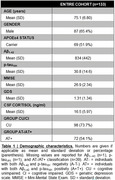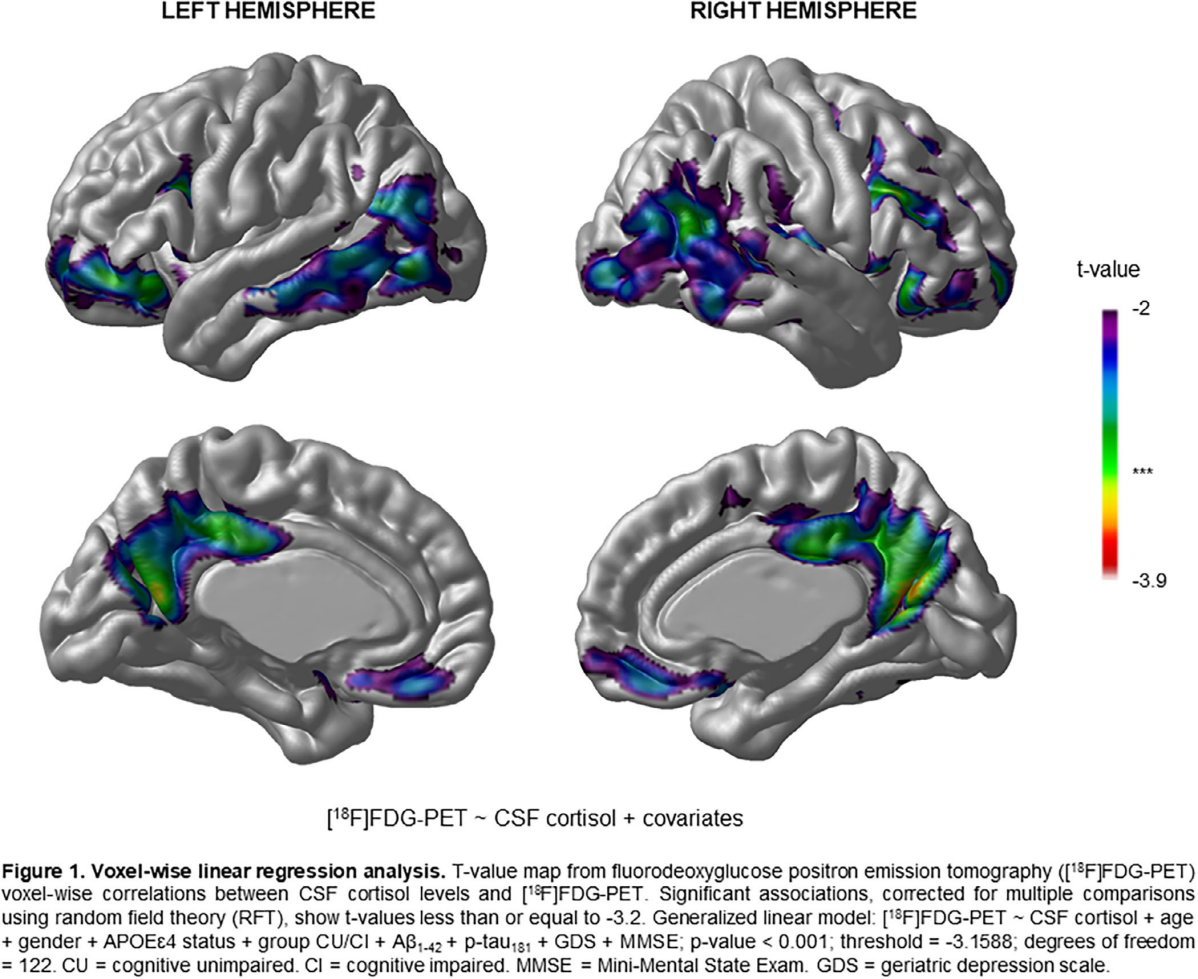# Association of CSF cortisol with brain glucose metabolism in Alzheimer's disease continuum

**DOI:** 10.1002/alz70856_106135

**Published:** 2026-01-07

**Authors:** Laura Willers Souza, Luiza Santos Machado, Guilherme Povala, Thomas Hugentobler Schlickmann, João Pedro Ferrari‐Souza, Marco Antônio De Bastiani, Lucas Uglione Da Ros, Andrei Bieger, Wyllians Vendramini Borelli, Guilherme Bauer‐Negrini, Jonathan M. DuBois, Eduardo R. Zimmer

**Affiliations:** ^1^ Universidade Federal do Rio Grande do Sul, Porto Alegre, Rio Grande do Sul, Brazil; ^2^ Department of Psychiatry and Neurochemistry, Institute of Neuroscience and Physiology, The Sahlgrenska Academy, University of Gothenburg, Gothenburg, VG, Sweden; ^3^ University of Pittsburgh, Pittsburgh, PA, USA; ^4^ Universidade Federal do Rio Grande do Sul, Porto Alegre, RS, Brazil; ^5^ Departments of Psychiatry and Neurology, University of Pittsburgh School of Medicine, Pittsburgh, PA, USA; ^6^ Neurology Department, São Lucas Hospital of PUCRS, Porto Alegre, Rio Grande do Sul, Brazil; ^7^ Centro de Memória, Hospital Moinhos de Vento, Porto Alegre, RS, Brazil; ^8^ Clinical Hospital of Porto Alegre, Porto Alegre, Rio Grande do Sul, Brazil; ^9^ Brain Institute of Rio Grande do Sul (InsCer), PUCRS, Porto Alegre, Rio Grande do Sul, Brazil; ^10^ Biogen, Cambridge, MA, USA; ^11^ McGill Centre for Studies in Aging, Montreal, QC, Canada

## Abstract

**Background:**

Elevated cortisol levels are considered a risk factor for Alzheimer's disease (AD). However, the relationship between cerebrospinal fluid (CSF) cortisol levels and AD pathophysiology remains insufficiently explored. Brain glucose hypometabolism, assessed with fluorodeoxyglucose positron emission tomography ([18F]FDG‐PET), is a hallmark of neurodegeneration in AD. Understanding the impact of CSF cortisol levels on brain metabolism is crucial for elucidating cortisol's role in the mechanisms underlying AD. This study aimed to investigate the association between CSF cortisol levels and [18F]FDG‐PET imaging across the biological and clinical continuum of AD.

**Method:**

We analyzed 133 participants from ADNI with available CSF cortisol measures, [18F]FDG‐PET imaging, and CSF Elecsys biomarkers (Aβ1‐42 and *p*‐tau181). Voxel‐wise linear regressions were conducted to evaluate the baseline associations between CSF cortisol levels and [18F]FDG‐PET using three models: (1) CSF cortisol as the independent variable; (2) interaction between CSF cortisol and clinical diagnoses (cognitive unimpaired and cognitive impaired); (3) interaction between CSF cortisol and AD biomarker positivity (Aβ1‐42 and *p*‐tau181 positivity). Models were adjusted for age, gender, APOEɛ4 status, cognitive status, mini‐mental state exam (MMSE) and geriatric depression scale (GDS) scores, and Aβ1‐42 and *p*‐tau181 levels. Results were corrected for multiple comparisons using random field theory (RFT, *p* < 0.001).

**Result:**

Demographics are depicted in Table 1. Voxel‐wise analyses identified a significant association between elevated CSF cortisol levels and brain glucose hypometabolism in several AD‐related brain regions, comprising the parietal lobe, lateral fronto‐orbital gyrus, right precuneus, frontal lobe, and inferior frontal gyrus (t‐value = ‐3.1588; Figure 1). Furthermore, no interactions were observed between CSF cortisol levels and clinical diagnoses or AD biomarker positivity in relation to brain metabolism (data not shown).

**Conclusion:**

This study demonstrated that CSF cortisol levels are associated with brain glucose hypometabolism in regions commonly vulnerable to AD. Notably, these associations were independent of AD biomarker positivity or clinical diagnoses. These findings suggest that elevated central cortisol levels may increase vulnerability in brain regions typically affected by AD, irrespective of the underlying pathophysiology. This underscores the potential role of cortisol as a risk‐related marker rather than a direct risk factor for AD, offering valuable insights into its contribution to disease mechanisms.